# Variable-selection ANOVA Simultaneous Component Analysis (VASCA)

**DOI:** 10.1093/bioinformatics/btac795

**Published:** 2022-12-10

**Authors:** José Camacho, Raffaele Vitale, David Morales-Jiménez, Carolina Gómez-Llorente

**Affiliations:** Signal Theory, Networking and Communications Department, University of Granada, Granada 18014, Spain; University of Lille, CNRS, LASIRE (UMR 8516), Laboratoire Avancé de Spectroscopie pour les Interactions, la Réactivité et l’Environnement, Lille F-59000, France; Signal Theory, Networking and Communications Department, University of Granada, Granada 18014, Spain; Department of Biochemistry and Molecular Biology II, School of Pharmacy, Institute of Nutrition and Food Technology “José Mataix”, Biomedical Research Center, University of Granada, Granada 18160, Spain; Instituto de Investigación Biosanitaria, ibs.GRANADA, Granada, Spain; CIBEROBN (Physiopathology of Obesity and Nutrition CB12/03/30038), Instituto de Salud Carlos III, Madrid 28029, Spain

## Abstract

**Motivation:**

ANOVA Simultaneous Component Analysis (ASCA) is a popular method for the analysis of multivariate data yielded by designed experiments. Meaningful associations between factors/interactions of the experimental design and measured variables in the dataset are typically identified via significance testing, with permutation tests being the standard go-to choice. However, in settings with large numbers of variables, like omics (genomics, transcriptomics, proteomics and metabolomics) experiments, the ‘holistic’ testing approach of ASCA (all variables considered) often overlooks statistically significant effects encoded by only a few variables (biomarkers).

**Results:**

We hereby propose Variable-selection ASCA (VASCA), a method that generalizes ASCA through variable selection, augmenting its statistical power without inflating the Type-I error risk. The method is evaluated with simulations and with a real dataset from a multi-omic clinical experiment. We show that VASCA is more powerful than both ASCA and the widely adopted false discovery rate controlling procedure; the latter is used as a benchmark for variable selection based on multiple significance testing. We further illustrate the usefulness of VASCA for exploratory data analysis in comparison to the popular partial least squares discriminant analysis method and its sparse counterpart.

**Availability and implementation:**

The code for VASCA is available in the MEDA Toolbox at https://github.com/josecamachop/MEDA-Toolbox (release v1.3). The simulation results and motivating example can be reproduced using the repository at https://github.com/josecamachop/VASCA/tree/v1.0.0 (DOI 10.5281/zenodo.7410623).

**Supplementary information:**

Supplementary data are available at *Bioinformatics* online.

## 1 Introduction

Designed experiments control the variation of one or more *factors* to assess the effect of such variation on one or more specific responses (variables) of interest. As an example, imagine that blood samples are extracted from two cohorts of cancer patients treated with two distinct drugs and characterized through chromatographic measures; one may then want to understand if and how the chromatographic profile of these blood specimens changes when altering the therapeutic strategy (i.e. the type of drug). If a single response is considered, the classical approach to identify those factors (e.g. the drug type) significantly influencing the observed variation is Fisher’s ANalysis of VAriance (ANOVA) (Fisher, 1919). To assess the significance of this influence, a given test statistic (e.g. the F-ratio) is used to compute Fisher’s *P*-value, which is then compared with a certain threshold for statistical significance. For multivariate responses (the current state of the art in, e.g. omics sciences like genomics, transcriptomics, proteomics or metabolomics), a widely adopted approach is to conduct multiple (univariate) tests over the individual variables, in an attempt to identify and select the specific responses significantly affected by a given experimental factor. A univariate test statistic (as in ANOVA) and its corresponding *P*-value is therefore obtained for each variable. Statistical significance thresholds in such multiple testing are typically adjusted (corrected) in order to identify as many significant variables as possible, while keeping the number of false positives under control according to certain criterion; well-known examples are the Bonferroni correction to control the family-wise error rate (FWER), as well as the Benjamini–Hochberg (BH) and Benjamini-Yekutieli procedures (Benjamini and Hochberg, 1995; Benjamini and Yekutieli, 2001) to control the false discovery rate (FDR). FDR-controlling procedures (or simply, *FDR methods*) are particularly appealing in multivariate settings with large numbers of variables due to their increased statistical power—they allow to identify more significant associations due to the less conservative *P*-value correction compared to, e.g. the Bonferroni correction. Thus, FDR methods—and particularly the BH procedure—have been widely used for multiple significance testing in omics data. However, as they are based on univariate tests, FDR methods do not take into account the possibly complex multivariate structure underlying the data at hand. Indeed, the effect of an experimental factor may often produce inter-related changes in multiple measured variables (e.g. a linear combination of responses), rather than simple variations within each individual one of them. This limits the detection power of the FDR approach.

An alternative strategy to treat multiple responses is to apply multivariate testing procedures, such as Multivariate ANOVA (MANOVA) (Warne, 2014). These methods replace multiple univariate tests by a single multivariate one to assess the statistical significance of the ANOVA model, including all the variables in the dataset. This avoids the need for multiple-testing corrections, but suffer from the effects of statistical noise, particularly when the number of variables is large, leading to a remarkable lack of discriminatory power. The multivariate test statistic often contains noisy contributions from a large number of (insignificant) variables, and factors affecting only a few of them can potentially be overlooked due to the overall statistical noise.

ANOVA Simultaneous Component Analysis (ASCA)—Smilde *et al.* (2005) and Jansen *et al.* (2005)—is one popular multivariate extension of ANOVA, widely employed in, e.g. chemistry, biology and biomedicine (Bevilacqua *et al.*, 2013; De Luca *et al.*, 2016; Du *et al.*, 2017; Firmani *et al.*, 2020; Nueda *et al.*, 2007; Smilde *et al.*, 2005). It combines the variance factorization and inference capabilities of ANOVA with the exploratory power of Principal Component Analysis (PCA). In ASCA, the statistical significance of factors’ and interactions’ effects is typically estimated by permutation testing (Anderson and Braak, 2003; Vis *et al.*, 2007): basically, the data variation induced by such effects is contrasted against an empirical null-distribution obtained through resampling. Albeit this strategy shows notable advantages compared to other testing approaches (Vis *et al.*, 2007), it inherits the limitations of multivariate testing. Indeed, the ‘holistic’ testing approach of ASCA (all variables considered at the same time) typically fails when trying to find any statistical significance for factors associated with only a reduced sub-set of variables.

In this article, we propose a generalization of ASCA by introducing a new method for variable selection, termed Variable-selection ASCA (VASCA). The main idea is to incorporate variable selection in the multivariate permutation testing procedure of ASCA to robustly assess the statistical significance of the experimental model. The proposed testing procedure attains improved detection power without compromising the Type-I error risk, while still being able to fully capture the inherent multivariate nature of the investigated data. The enhanced statistical power brought by variable selection leads to improved ASCA modeling; for a given effect (factor/interaction), VASCA identifies significant associations with reduced sub-sets of variables, filtering out those not accounting for the effect itself, and narrowing down the subsequent ASCA analysis to a limited amount of meaningful responses.

VASCA is here assessed with simulations and with a real dataset from a multi-omic clinical experiment, and compared to ASCA and the BH (FDR) method in terms of statistical power, and to partial least squares discriminant analysis (PLS-DA) and its sparse counterpart (sPLS-DA) in terms of exploratory power. We also include a comparison with an early approach for variable selection in ASCA, the ASCA-genes method by Nueda *et al.* (2007).

## 2 ANOVA Simultaneous Component Analysis

The most established ASCA pipeline, consistent with that of ANOVA, is based on several steps: (i) factorization of the data according to the factors/interactions of the experimental design under study; (ii) significance testing (based on permutation tests) for factors/interactions; (iii) visualization of significant factors’/interactions’ effects using Principal Component Analysis (PCA) to understand separability among levels and, optionally, *post hoc* testing of levels using confidence intervals. The interested reader can find an example of all these data analysis steps in the Supplementary Material. The code for reproducing this example is included in the aforementioned online repository. A detailed description of the ASCA modeling framework is provided next.

### 2.1 Factorization of the data

Let **X** be an *N *×* M* data matrix with *N* the number of conducted trials and *M* the number of responses or variables recorded in a series of designed experiments. For the sake of simplicity and without loss of generality, we will consider the case of a design encompassing two fixed factors. The data in **X** can be decomposed as

(1) $$X=1m^{T}+A+B+AB+E,$$

where 1 is a vector of ones of suitable length (N×1), **m** (M×1) denotes a vector containing the mean values of all the *M* measured variables, while **A** and **B** represent the factor matrices, **AB** the interaction matrix and **E** the residual matrix, all of size *N *×* M*. The idea behind this decomposition is to partition the variation in the dataset **X**, according to the different factors/interactions of the design. In this article, we use the technique referred to as ASCA+ (Thiel *et al.*, 2017) to account for mild unbalancedness in the data. Basically, the decomposition is derived as the least squares solution of a regression problem, where **X** is regressed onto a coding matrix **C** as:

(2) $$X=C\Theta +E=1\theta_{m}+C_{A}\Theta_{A}+C_{B}\Theta_{B}+C_{AB}\Theta_{AB}+E,$$

where $C=[1,C_{A},C_{B},C_{AB}]$ is built from the design matrix using the sum coding or deviation coding approach (Thiel *et al.*, 2017). Namely:

-
1 is a vector of ones of suitable length;
- for a generic factor *Z*, the corresponding vector/matrix $C_{Z}$ has dimensions $N\times (L_{Z}-1)$, with *L_Z_* the number of levels of the tested factor. If the *n*th observation in **X** was collected at one of the first $L_{Z}-1$ levels of *Z*, then $C_{Z}$ contains a one in the *n*th row of its $L_{Z}-1$th column. The rows of $C_{Z}$ corresponding to the observations in **X** recorded at the last level of *Z*, instead, carry –1 in all their entries. The remaining elements of $C_{Z}$ equal 0;
- for a given interaction *ZY*, the matrix $C_{ZY}$ has size $N\times (L_{Z}\cdot L_{Y})$ and results from the pair-wise multiplication of the columns of **C** associated to the factors *Z* and *Y*.

Take for instance the following example, where we assess a response of a system at three different levels of temperature and two levels of pH. Thus, we have two factors, with three and two levels, respectively, in the design matrix **F**, where we also include two replicates per testing condition. The corresponding coding matrix **C** when considering the factors and their interaction contains five columns: two for the first factor, where 30°C is coded with minus ones, one for the second factor and two for the interaction, computed by multiplying each column of the first factor by the single column of the second factor:

(3) $$\begin{array}{ccc} F=\left[\begin{array}{cc} 10{}^{\circ}C & 4.0\\ 10{}^{\circ}C & 4.0\\ 20{}^{\circ}C & 4.0\\ 20{}^{\circ}C & 4.0\\ 30{}^{\circ}C & 4.0\\ 30{}^{\circ}C & 4.0\\ 10{}^{\circ}C & 6.0\\ 10{}^{\circ}C & 6.0\\ 20{}^{\circ}C & 6.0\\ 20{}^{\circ}C & 6.0\\ 30{}^{\circ}C & 6.0\\ 30{}^{\circ}C & 6.0 \end{array}\right] & & C=\left[\begin{array}{ccccccc} 1 & 0 & | & 1 & | & 1 & 0\\ 1 & 0 & | & 1 & | & 1 & 0\\ 0 & 1 & | & 1 & | & 0 & 1\\ 0 & 1 & | & 1 & | & 0 & 1\\ -1 & -1 & | & 1 & | & -1 & -1\\ -1 & -1 & | & 1 & | & -1 & -1\\ 1 & 0 & | & -1 & | & -1 & 0\\ 1 & 0 & | & -1 & | & -1 & 0\\ 0 & 1 & | & -1 & | & 0 & -1\\ 0 & 1 & | & -1 & | & 0 & -1\\ -1 & -1 & | & -1 & | & 1 & 1\\ -1 & -1 & | & -1 & | & 1 & 1 \end{array}\right] \end{array}.$$

Thus, in Eq. (2), $C_{A},\;C_{B}$ and $C_{AB}$ represent the coding for factors A and B and their interaction AB, respectively, and $\Theta =[\theta_{m},\Theta_{A},\Theta_{B},\Theta_{AB}]$, is composed of the coefficients of the factorization estimated by least squares:

(4) $$\Theta ={\left(C^{T}C\right)}^{-1}C^{T}X.$$

This solution minimizes the variance in the residual matrix **E**, which is obtained as

(5) E=X−CΘ.

### 2.2 Statistical significance testing

Just like in ANOVA, for inference after the data decomposition of **X**, we test the statistical significance of the individual factors and interactions. Thus, we can determine which of them have a significant impact on the multivariate responses in **X**. A widely used approach for ASCA inference is permutation testing.

Permutation testing in the context of ASCA can be performed by randomly shuffling the rows of **X** in Equation (4), yielding a new set of regression coefficients:

(6) $$\Theta^{*}={\left(C^{T}C\right)}^{-1}C^{T}X^{*},$$

where ${}^{*}$ stands for permuted. Then, the permuted factorized data for any factor/interaction *Z* is re-computed as $Z^{*}=C_{Z}\Theta_{Z}^{*}$ and the error as $E^{*}=X^{*}-C\Theta^{*}$. Analogously, one can permute the rows or values in **C** instead of those in **X** (Camacho *et al.*, 2022).

Permutation tests are carried out by comparing a given statistic, computed after the ASCA factorization, with the corresponding statistic computed from hundreds or more permutations. The *P*-value is obtained as (Throughout the article, we assume that the higher the statistic the more significant the effect of the factor/interaction.)

(7) $$p=\frac{\#\{S_{k}^{*}\geq S;k=1,\ldots ,K\}+1}{K+1},$$

where *S* refers to the statistic computed from the factorized data matrix, $S_{k}^{*}$ is the statistic corresponding to the *k*th random permutation, #{cond} refers to the number of times condition *cond* is met, and *K* is the total number of permutations. Thus, the *P*-value yields an empirical estimate of the probability of obtaining a result as (or more) extreme as the observed one when the null hypothesis holds.

There are several choices for the ASCA test statistic—see Camacho *et al.* (2022) for a recent review on the permutation approach and the relevance of the chosen statistic. The (Type-I) sum-of-squares of the factor/interaction matrix $||Z|{|}_{F}^{2}$ was proposed as the original ASCA statistic (Vis *et al.*, 2007). Motivated by the data visualization aspect of ASCA, which typically uses the first two Principal Components (PCs), Zwanenburg *et al.* (2011) proposed testing the sum-of-squares of the first two PCs of the factor/interaction matrix. This is also used by Thiel *et al.* (2017). More recent variants of ASCA (Marini *et al.*, 2015; Martin and Govaerts, 2020) employ the F-ratio, computed as the ratio of the mean sum-of-squares of the factor/interaction and the suitable next order factor/interaction (often the residuals)—see Anderson and Braak (2003) for a detailed discussion of the orders of factors and interactions in complex designs. Finally, in ASCA extensions for unbalanced data (Martin and Govaerts, 2020; Thiel *et al.*, 2017), the utilization of the Type-III sum-of-squares is proposed for testing purposes; this is computed from the difference between the residuals in the reduced and full models (see references above for more detail).

### 2.3 Visualization and *post hoc* tests

Significance testing in ANOVA/ASCA reveals the statistical significance of factors’ and interactions’ effects, but not the specific levels (or combination of levels) that are actually associated to significant differences in the responses. To identify significant differences across levels, *post hoc* tests (carried out using PCA in the context of ASCA) are typically employed.

Several visualization methods have been proposed that combine the ANOVA-like decomposition in Equation (2) with subspace visualization, in particular with PCA. Among these, ASCA and ANOVA-PCA (APCA) are closely related (Thiel *et al.*, 2017; Zwanenburg *et al.*, 2011) and both share the same approach for factorization and inference (significance testing), prior to visualization. For visualization and *post hoc* testing, APCA performs PCA on each significant factor/interaction matrix plus the unexplained variance: Z+E. ASCA, however, carries out PCA on **Z**, and then displays the projection of Z+E onto the resulting loadings in what is known as a scores plot. As a consequence, for each factor/interaction, we can compute as many PCs as the corresponding degrees of freedom. In both APCA and ASCA, the score-plot is used for the visualization and possible significance testing (Liland *et al.*, 2018) of the differences among levels.

## 3 VASCA

VASCA is based on breaking down the single test statistic computed in ASCA (all variables considered), i.e. *S* in Eq. (7), into variable-wise statistics *S^v^* for v∈{1,..,M}. This approach can be generalized to any test statistic based on sums-of-squares (see Section 2.2), which can be computed for each variable independently. Since the F-ratio is also based on (mean) sums-of-squares, the computation of variable-wise F-ratios is also possible.

VASCA starts by sorting out the measured variables in decreasing order of *S^v^*. Then, it sequentially assesses the statistical significance of the data matrix composed of the ensemble of the first *m* variables in this ordering, with *m* ranging from 1 to *M*. The null-hypothesis is rejected for the largest significant data matrix, (Notice that this does not mean that all variables in this matrix are statistically significant, but that their multivariate combination is. We will illustrate this difference with simulated examples.) In this regard, variable selection in VASCA resembles the step-up BH procedure (Benjamini and Hochberg, 1995) and generalizes ASCA, since it includes the significance assessment for the entire dataset with *M* variables. For this reason, VASCA is at least as powerful as ASCA. Moreover, both ASCA and VASCA generalize ANOVA, since (V)ASCA applied to an individual response variable boils down to an ANOVA based on permutation testing, thus, robust to deviations from the normal distribution (Anderson and Braak, 2003).

Let us define:

(8) $$\Phi_{m}=\underset{\left\{v_{1},\ldots ,v_{m}\}\in \{1,\ldots ,M\right\}}{\text{argmax}}\sum_{v=v_{1},\ldots ,v_{m}}S^{v}$$

as the sub-set of *m* variables $\Phi_{m}=\{v_{1},\ldots ,v_{m}\}$ that maximizes the sum statistic $\sum_{v\in \Phi_{m}}S^{v}$. We refer to $S^{\Phi_{m}}$ as the corresponding multivariate statistic for this sub-set, i.e. the statistic computed from the whole data (sub)matrix rather than from individual variables.

Testing for significance in VASCA requires careful consideration of the permutations across the selected variables. This is key to provide a meaningful null-distribution and to guarantee the statistical power of the approach. Rather than using multiple-testing corrections like in the BH procedure, we embed the variable-selection mechanism within the permutation testing. Within each permutation *k* we reorder the variables in decreasing order of the permuted, variable-wise statistic ${\left(S^{v}\right)}_{k}^{*}$. For a given number of variables *m*, the aforementioned sub-set of variables after the permutation is recomputed as ($\Phi_{m}{)}_{k}^{*}=\text{arg}{\text{max}}_{v_{1},\ldots ,v_{m}}\sum_{v=v_{1},\ldots ,v_{m}}{\left(S^{v}\right)}_{k}^{*}$, so that ${\left(\Phi_{m}\right)}_{k}^{*}$ will most likely contain different variables from those in $\Phi_{m}$. Then, we recompute the corresponding statistic ${\left(S^{\Phi_{m}}\right)}_{k}^{*}$. The set of statistics ${\left(S^{\Phi_{m}}\right)}_{k}^{*}$ for different permutations *k* (and sub-sets of *m* variables) characterizes the null-distribution of the VASCA testing strategy and is employed to compute the following *P*-value for statistical significance:

(9) $$p_{m}=\frac{\#\{{\left(S^{\Phi_{m}}\right)}_{k}^{*}\geq S^{\Phi_{m}};k=1,\ldots ,K\}+1}{K+1}$$

This means that in any given sub-set of *m* variables, significance is assessed by contrasting the true $S^{\Phi_{m}}$ with the permuted ${\left(S^{\Phi_{m}}\right)}_{k}^{*}$, where the specific *m* variables may not be the same. For some statistics, this may require some form of normalization. For example, if we use $||Z|{|}_{F}^{2}$ as the test statistic, a variable with higher variance in the raw data may likely show higher $||Z|{|}_{F}^{2}$. However, for a sound comparison within the context of permutation testing, we need all variables to exhibit similar expected test statistic values under the null hypothesis. For this reason, in this article, when we use $||Z|{|}_{F}^{2}$ as the test statistic, we auto-scale (normalize to 0 mean and unit variance) the data. The F-ratio (whose utilization will be more frequent in this work) does not need such normalization, since it is normalized by definition.

Given that we select the maximum *m* for which significance is found, i.e. max(m) with *p_m_* below the significance threshold, and due to the multivariate nature of VASCA, (sub)matrices called significant may indeed include some non-significant variables. This is due to the filtering nature of multivariate models and may be seen as a disadvantage over univariate tests such as BH (FDR). However, from the set of *m* selected variables, a subsequent analysis based on PCA can help distinguishing variables that are truly relevant from those that are not. We can also employ a bootstrapping procedure to identify PCA loadings (variables) with values significantly different from 0. Thus, all variables corresponding to non-significant loadings can be discarded *a posteriori*.

### 3.1 Connection with the ASCA-genes method

The closest approach to VASCA reported in literature is the ASCA-genes method by Nueda *et al.* (2007). ASCA-genes identifies relevant variables with permutation testing, but with two main differences with respect to VASCA:

- First, ASCA-genes is based on the evaluation of two indices borrowed from the Multivariate Statistical Process Control (MSPC) domain (Ferrer, 2007): model leverage and squared prediction error (SPE)—see Nueda *et al.* (2007) for details on their mathematical definition and practical interpretation. Briefly speaking, most relevant variables are expected to show a high leverage and a low SPE. Variables with high SPE and leverage are considered as poorly modeled but still potentially interesting. High SPE and low leverage variables are deemed as odd. Variables exhibiting low leverage and low SPE are regarded as not relevant. In order to compute leverage and SPE, though, ASCA-genes require factors/interactions with at least two degrees of freedom, i.e. factors with at least three levels or interactions involving at least a factor with three levels. Conversely, VASCA computes a single statistic from any matrix resulting from the ANOVA factorization of **X**, which simplifies interpretation and it is more flexible (i.e. it can be applied to factors/interactions with only one degree of freedom).
- Second, and most importantly, ASCA-genes is based on a regular permutation approach, while VASCA is grounded on the reordering of the variables within each permutation. This is key to avoid false positives.

## 4 Evaluation in simulation examples

We devised four simulated experiments in order to compare VASCA with ASCA and ASCA-genes, and the BH procedure to control the FDR, simply referred to as FDR from now on. For simplicity and for the sake of clarity, in the first three examples we simulate a single factor with two levels, where each level includes 20 subjects (thus, a total of 40) for which 400 variables are collected. This choice is motivated by the nature of typical omics experiments (Tenorio-Jiménez *et al.*, 2019). In the first and the last examples, we consider a two factor/multi-level problem, in which ASCA-genes can be applied thanks to the increased number of degrees of freedom.

To simulate the background in the multivariate data **X**, we use the SimuleMV tool (Camacho, 2017) available together with ASCA and VASCA in the MEDA Toolbox at https://github.com/josecamachop/MEDA-Toolbox. SimuleMV allows to simulate a dataset with a certain level of correlation. The inputs to SimuleMV are the size of the data matrix (number of rows and columns) and a level of correlation between 0 (absence) and 10 (maximum correlation). In our simulations, we chose Levels 7 and 8. Each simulated experiment is repeated 1000 times and average and standard deviation results are presented for the *P*-values, along with Type I and Type II error rates.

For comparison purposes, FDR was implemented from the (empirical) probability distribution obtained through permutation testing, and corrected *P*-values were computed through the BH procedure. In all cases, the permutations used the same seed for the random generation engine in the four methods, ASCA, FDR, ASCA-genes and VASCA. Each *P*-value in any of the methods is computed with 1000 permutations.

### 4.1 Example 1: Non-significant relationship

The first example illustrates the case where the data matrix **X** and the class coding **C** for the factor are unrelated. We generate **X** with the SimuleMV tool. The design matrix **F** is obtained in two steps: first, we draw 40 observations *l_i_* from a normal distribution with zero mean and standard deviation one; second, we assign each of the 40 rows of **X** to one of the classes depending on the sign of the corresponding observation *l_i_*. Finally, we construct **C** from **F** using deviation coding (as discussed in Section 2.1). In summary:

$$\begin{array}{c} X\leftarrow \mathrm{SimuleMV}(40,400,7)\\ F\leftarrow \mathrm{sign}(r),\;\text{with}\;r∼N(0,1)\\ C\leftarrow \mathrm{dev}\_\mathrm{cod}(F) \end{array}$$

We repeat this data generation procedure 1000 times. Given the independent generation of **X** and **C**, we expect no statistical significance to be found in the analysis by any of the methods. Results obtained using the F-ratio as test statistic are shown in Supplementary Figure S5 (similar results were obtained using the sum-of-squares as test statistic). Supplementary Figure S5 depicts the ordered *P*-values obtained for FDR and VASCA, and the single *P*-value (for the complete matrix **X**) for ASCA. Average results are shown with the corresponding lines and the shadowed areas represent standard deviations. Typically considered thresholds for statistical significance at 0.05 and 0.01 are also included as control limits. Note that vertical axes are in logarithmic scale.

We can see that VASCA and ASCA generally converge in this example, and that all methods yield *P*-values well above the control limits, illustrating their robustness against Type-I errors. QQ-plots for ASCA and VASCA (Supplementary Fig. S6) show that the *P*-values are uniformly distributed under the null-hypothesis, as expected, while the empirical false positive rate (FPR) (Table 1) is very close to the significance level of 0.01 for both ASCA and VASCA and is almost identical regardless of the chosen test statistic. The FDR, however, attains an FPR much lower than the significance level, as a result of the BH correction procedure—which aims to control the FDR at the significance level, and results in too conservative corrections in terms of the FPR in this case. Similar results are obtained for **X** drawn at random from a multinormal distribution, rather than using the SimuleMV tool (These results can be found in the software repository.).

**Table 1.** Example 1: Type-I error measured as false positive rate (FPR) for FDR, ASCA and VASCA using the sum-of-squares (SSQ) and the F-ratio as test statistics for a significance level of 0.01

Method	FPR (SSQ)	FPR (F-ratio)
FDR	9.3×10^–5^	9.3×10^–5^
ASCA	0.01	0.01
VASCA	0.0108	0.0108

A major strength of ANOVA, also inherited by ASCA, VASCA or the FDR, is the possibility to analyze complex experimental designs with several factors and interactions. In a second experiment we simulate datasets with two factors, of four and three levels, respectively, according to a balanced full-factorial design with four replicates. This design provides a total number of 4×3×4=48 experiments (rows). **X** is simulated as in the previous example, independently from the factors, so that no significant association is expected. With this example, we can compare VASCA to ASCA-genes in terms of Type-I error. The simulation follows (We implicitly assume that **C** is built from **F** using deviation coding.):

$$\begin{array}{c} X\leftarrow \mathrm{SimuleMV}(40,400,7)\\ F\leftarrow \mathrm{FullFactorial}(4,3,4) \end{array}$$

The empirical FPR is presented in Table 2. We see that ASCA-genes is slightly over optimistic in comparison to VASCA.

**Table 2.** Example 1b: Type-I error measured as false positive rate (FPR) for FDR, ASCA, VASCA and ASCA-genes using the F-ratio as test statistics for a significance level of 0.01

Method	FPR (factor 1)	FPR (factor 2)
FDR	2.3×10^–4^	1.4×10^–4^
ASCA	0.017	0.007
VASCA	0.015	0.008
ASCA-genes	0.024	0.025

From this simulation, we can conclude that when the responses in **X** and the factors in **C** are independent, we obtain a uniform distribution of the *P*-values in ASCA and VASCA which conveniently adjusts the Type-I error to the significance level.

### 4.2 Example 2: Significant one-to-one relationships

We start by generating **F** like in the first example. We then simulate **X** with the SimuleMV tool except for the first three variables that are drawn from a normal distribution with zero mean and unit standard deviation. We thus make these three variables independent from the rest and no spurious correlation is induced with the other variables in **X**. Then, we modify these three variables so that a significant bias is induced between observations from the two classes.

$$\begin{array}{c} F\leftarrow \mathrm{sign}(r),\;\text{with}\;r∼N(0,1)\\ X_{i}\leftarrow r_{i}+5\cdot C,\;\text{with}\;r_{i}∼N(0,1);\;i=1,2,3\\ X_{j}\leftarrow \mathrm{SimuleMV}(40,397,7);\;j=4,\ldots ,400 \end{array}$$

We repeat this data generation procedure 1000 times. The simulation is designed to generate a one-to-one relationship between each of the three variables and the simulated factor in **C**. We expect both the FDR method and VASCA to identify these relationships, but because they are only present in 3 out of the 400 variables, we expect ASCA to overlook it.

**Fig. 1.**
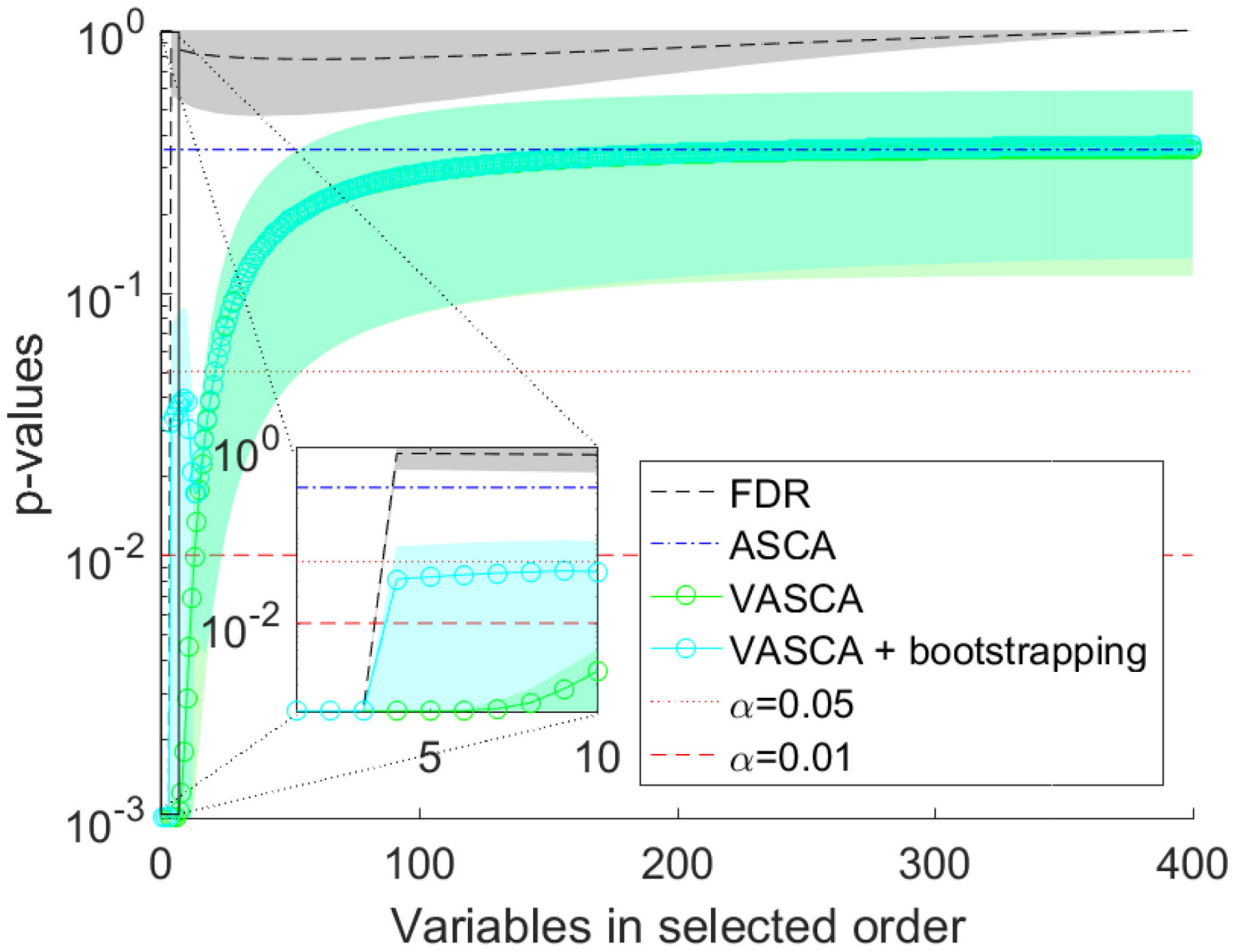
Example 2: One-to-one relationships between three variables in **X** and **C**. Comparison of *P*-values computed with FDR, ASCA and VASCA (without and with bootstrapping). For each method, average *P*-values from 1000 simulations are shown together with a shaded area corresponding to one standard deviation. For the FDR, we represent the *P*-values in increasing order from left to right (from the most to the least significant variable), corrected following the procedure of BH. Whenever a corrected *P*-value exceeds 1, a value of 1 is used instead. For ASCA, a single *P*-value is shown, corresponding to the *P*-value for the dataset with 400 variables averaged over the 1000 simulations. For VASCA, the *P*-value at each number of variables *m* represents the significance of the dataset including the most significant *m* variables. The inset represents a detail for the first (most significant) 10 variables. Control limits of statistical significance (α=0.05 and α=0.01) are also represented

Results are presented in Figure 1. As expected, FDR and VASCA systematically identify the one-to-one relationships (*P*-value < 0.001 for the first variables), while ASCA identifies the factor as non-significant, well above the control limits. Again, we can see that VASCA exactly matches ASCA for the complete set of 400 variables, since the former generalizes the latter. This example illustrates the variable selection capability of VASCA as a clear advantage over ASCA.

The zoomed image in Figure 1 reflects that VASCA identifies models up to 6–10 variables as significant (*P*-value < 0.001 for 6 variables, *P*-value < 0.01 for 10). Contrarily, FDR accurately identifies only three variables as truly significant. The outcome of VASCA is a consequence of the filtering nature of multivariate models, i.e. models with 6/10 variables are called significant, even when only 3 of these variables are truly associated with the factor. Significance results are however refined in the subsequent analysis step of VASCA. To see this, let us proceed with the workflow of VASCA, by visualizing the factorized data with PCA in Supplementary Figure S7. The scores, shown in panel A, clearly distinguish the two classes, confirming the significance of the model (The example encompasses a factor of two levels which makes the factorized matrix in ASCA of rank one, and only one PC can be extracted.). The loadings in panel B show that three variables are by far the most relevant of the six under study (shown by the magnitude of the loadings). The bootstrapping intervals indicate that only those three variables are statistically significant (loadings significantly different from 0). We applied the same bootstrap approach to the 1000 repetitions in the simulation, to depict a curve of significance for VASCA + bootstrapping in Figure 1, showing the same accuracy as the FDR method at a *P*-value threshold of 0.01. In Supplementary Table S1 we show the percentage of simulations where at least 1, 2 and 3 of the significant variables were found to be statistically significant along with the FPR. The results are satisfactory for the three compared methods, although VASCA shows an increase in the FPR if we compute it including all variables in significant matrices. If we apply bootstrapping along with VASCA, we reduce the FPR to a reasonable (but still slightly overoptimistic) level.

Finally, we wanted to check what would happen if we repeat the same simulation scheme of the example, but with a much smaller bias in the three variables, so that the variance that reflects the connection between them and the factor is 10 times smaller than in the previous case. For this purpose, we recompute significant responses as:

$$X_{i}\leftarrow r_{i}+0.5\cdot C,\;\text{with}\;r_{i}∼N(0,1);\;i=1,2,3$$

and repeat the simulation. The results are shown in Supplementary Figure S8 and Supplementary Table S2. We can see that VASCA (with and without bootstrapping) outperforms the FDR in terms of statistical power. Even without bootstrapping, VASCA adequately controls the FPR (the FPR inflation issue encountered in the previous example uniquely arises when only some variables in **X** are clearly significant). Therefore, VASCA does not identify fake isolated biomarkers, but in a set of discovered biomarkers, it may include a subset of not truly significant ones (and we may still detect this through visualization or bootstrapping). The resulting benefit is an increased statistical power, which in this case is the result of VASCA taking advantage of the underlying correlation among the three variables associated to the factor.

### 4.3 Example 3: Multivariate relationship

In this example, we generate again **X** with the SimuleMV tool except for three variables that are drawn from a normal distribution with mean zero and standard deviation one. We thus make these three variables independent from the rest and no spurious correlation is created between **C** and **X**. **F** is obtained in two steps: we first obtain 40 values *l_i_* by summing the three normal variables in **X**; then, we assign each of the 40 rows of **X** to one of the design levels depending on the sign of the corresponding sum. With this approach, we have created an additive multivariate relationship between the three variables in **X** and **C**. Therefore, unlike the previous example, we need to consider the combination of the three variables to properly differentiate the aforementioned levels. Additive multivariate relationships are consistent with the interpretation of biomarkers in networks of pathways, where correlations are identified as paths that jointly contribute to a response/reaction.

$$\begin{array}{c} X_{i}∼N(0,1);\;i=1,2,3\\ X_{j}\leftarrow \mathrm{SimuleMV}(40,397,7);\;j=4,\ldots ,400\\ F\leftarrow \mathrm{sign}(X_{1}+X_{2}+X_{3}) \end{array}$$

**Fig. 2.**
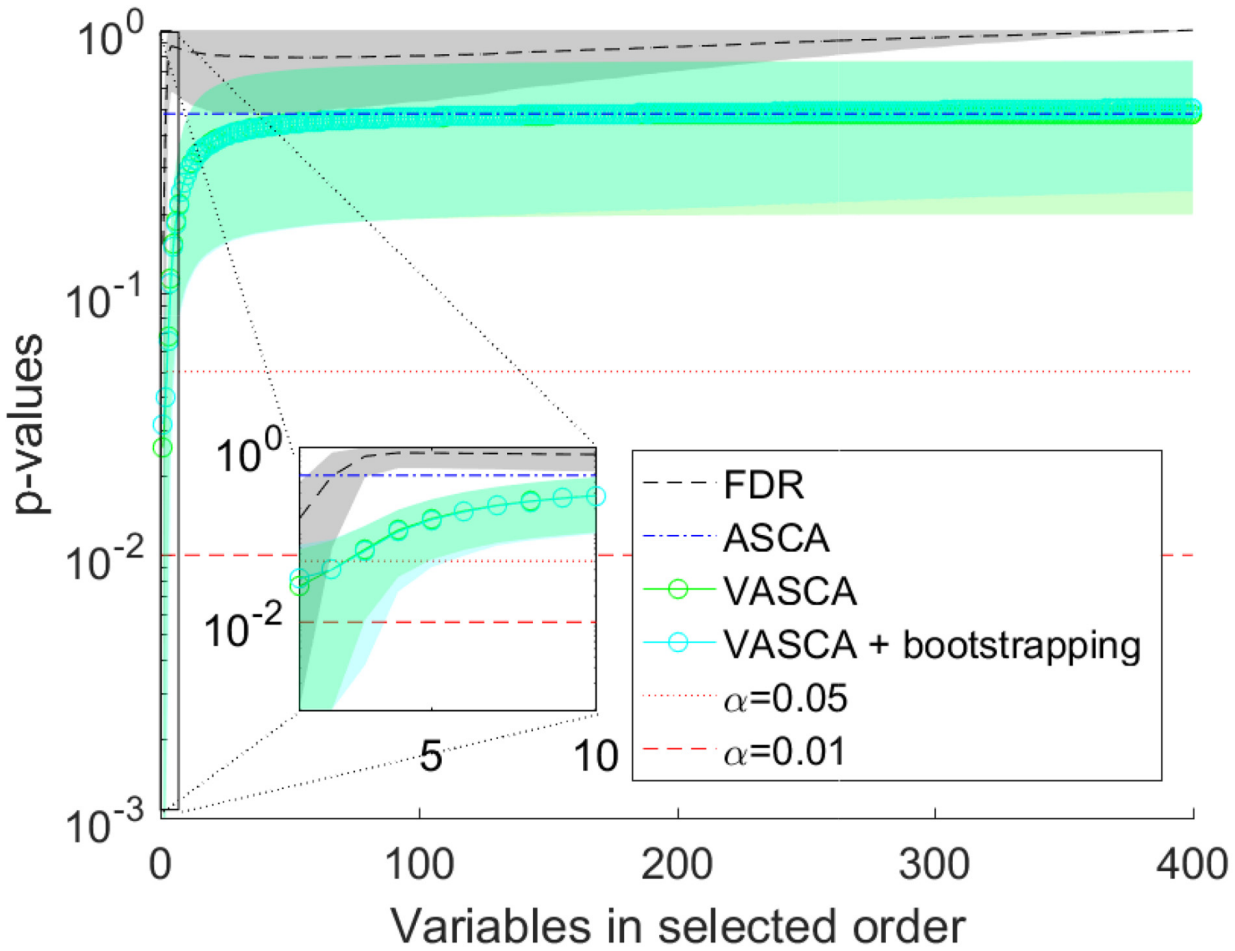
Multivariate relationship between three variables in **X** and **C**. Comparison of *P*-values computed with FDR, ASCA and VASCA (without and with bootstrapping). For each method, average *P*-values from 1000 simulations are shown together with a shaded area corresponding to one standard deviation. For the FDR, we represent the *P*-values in increasing order from left to right (from the most to the least significant variable), corrected following the procedure of BH. Whenever a corrected *P*-value exceeds 1, a value of 1 is used instead. For ASCA, a single *P*-value is shown, corresponding to the *P*-value for the dataset with 400 variables averaged over the 1000 simulations. For VASCA, the *P*-value at each number of variables *m* represents the significance of the dataset including the most significant *m* variables. The inset represents a detail for the first (most significant) 10 variables. Control limits of statistical significance (α=0.05 and α=0.01) are also represented

Results are presented in Figure 2 and Supplementary Table S3. The results show the increased power of VASCA over the FDR in a similar way as in the previous example. Again, VASCA without bootstrapping adequately controls the FPR. ASCA once again overlooks the relationship. This example illustrates the benefits brought by the multivariate nature of VASCA which, as opposed to the FDR method, is able to identify multivariate additive relationships among variables.

### 4.4 Example 4: Multivariate relationship in two factors and interaction with several levels

In this last experiment we simulate datasets with two significant factors, of four and three levels, respectively, once again according to a balanced full-factorial design with four replicates. As in the previous example, a significant multivariate relationship exists between each of the factors and three variables.

$$\begin{array}{c} F\leftarrow \mathrm{FullFactorial}(4,3,4)\\ v_{i}(l_{1})∼N(0,1);\;l_{1}=1,2,3,4\;i=1,2,3\\ v_{i}(l_{2})∼N(0,1);\;l_{2}=1,2,3\;i=1,2,3\\ X_{i}(l_{1},l_{2})\leftarrow \mathrm{SimuleMV}(4,3,8)+0.5v_{i}(l_{1})+0.5v_{i}(l_{2});\;i=1,2,3\\ X_{j}\leftarrow \mathrm{SimuleMV}(40,397,7);\;j=4,\ldots ,400 \end{array}$$

Results are presented in Supplementary Figure S9 and Table 3. We see that VASCA (with and without bootstrapping) outperforms the FDR and ASCA-genes in terms of statistical power. Like in Section 4.3, even without bootstrapping, VASCA adequately controls the FPR. ASCA-genes shows an excess in the Type I error (FPR) but for a reduced power.

**Table 3.** Example 4: multivariate relationship between 3 variables in **X** and **C** for a design with 2 factors of 4 and 3 levels, respectively

Method	1 variable	2 variables	3 variables	FPR
FDR	0.849	0.517	0.157	1.6×10^–4^
FDR	0.832	0.446	0.088	2.0×10^–4^
ASCA-genes	0.613	0.201	0.024	0.022
ASCA-genes	0.778	0.382	0.072	0.020
VASCA	0.903	0.798	0.511	0.014
VASCA	0.895	0.759	0.412	0.017
VASCA + bootstrap	0.888	0.761	0.39	0.008
VASCA + bootstrap	0.886	0.687	0.237	0.007

*Note*: Proportion of simulations where at least 1, 2 and 3 of the truly significant variables (as predefined in the experiment) were found statistically significant by the different methods, and Type-I Error measured as false positive rate (FPR). Comparison of FDR, VASCA (without and with bootstrapping) and ASCA-genes for a significance level of 0.01.

For further comparison, we illustrate the result obtained by the ASCA-genes method by Nueda *et al.* (2007) in one repetition of the simulation—note that previous results showing *P*-values and Type-I and Type-II errors are averaged over 1000 repetitions. Results obtained with ASCA-genes in the 2-factor (4 and 3 levels) example of the present section can be found in Supplementary Figure S10. Two of the significant variables (marked in dark color) for Factor 1 show high leverage. The other significant variable is found in the SPE. For factor 2, however, there are several non-significant variables that exceed the leverage control limit, which should be regarded as false positives. In this specific example, VASCA detected two significant variables for Factor 1 (the third presented a *P*-value close to 0.05) and none for Factor 2, FDR could only detect one significant variable for Factor 1, and VASCA + bootstrap none. We can see that VASCA and ASCA-genes yield similar results, with VASCA being more general (i.e. it can be applied to rank-one factor/interaction matrices) and with a better compromise between Type I and Type II error risk.

## 5 Results on real data

The BIOASMA dataset (Gomez-Llorente *et al.*, 2020) comprises clinical, biochemical, anthropometrical parameters, inflammatory biomarkers, metagenomic and metabolomic data for 46 children (12 girls and 34 boys, aged 4–13 years) with an allergic asthma diagnosed based on the Spanish Guidelines for Asthma Management (GEMA criteria 4.4)—(Moral *et al.*, 2016). The children were also classified into normal-weight (*n* = 13), overweight (*n* = 8) and obese (*n* = 25) according to the age and sex-specific thresholds proposed by Cole *et al.* (2000). Biochemical data were obtained by routine methods. Inflammatory biomarkers were determined by ELISA and by XMAP Luminex technology. Metabolomic data were obtained by one-dimensional proton nuclear magnetic resonance (1D ^1^H-NMR) spectra of blood plasma samples. Short chain fatty acids were determined by Gas Chromatography-Mass Spectrometry. Metagenomic data were obtained by 16sRNA barcoding sequencing and the Amplicon sequence variants (ASVs) were normalized by the rarefaction method (Gotelli and Colwell, 2001). Deriving potential biomarkers from this dataset represents a real challenge (Gomez-Llorente *et al.*, 2020), given the low sample size and the complexity of the experimental design: two potential conflicting factors (asthma severity and weight classification/status) with three levels each are taken into account and the individuals distribution is significantly unbalanced.

### 5.1 Factor-wise models

The statistical results obtained from the original analysis of the dataset (Gomez-Llorente *et al.*, 2020) are based on PLS-DA (Barker and Rayens, 2003) and its sparse variant sPLS-DA (Lê Cao *et al.*, 2008). sPLS-DA considers variable selection during model calibration with the idea of discarding non-informative variables. Neither PLS-DA nor sPLS-DA models were statistically significant to distinguish the three classes of weight status or the three classes of asthma severity. However, for individual sPLS-DA models for both factors it was possible to find statistically significant differences between one of the classes versus the rest. In particular, a sPLS-DA model with 12 variables was found statistically significant to distinguish the persistent asthma class from the rest (occasional and frequent asthma) with an Area Under the Receiver Operating Characteristics curve (AUROC) of 0.66 ± 0.08 (*P*-value < 0.05) in double cross-validation (Szymańska *et al.*, 2012), and a sPLS-DA model with three variables was found statistically significant to distinguish the normo-weight class from the rest (overweight and obese) with an AUROC of 0.75 ± 0.09 (*P*-value < 0.05). The PLS-DA version of the first model (asthma severity) with only selected variables is presented in Supplementary Figure S11, where the scores show a clear separation between persistent asthma and the rest. The model for weight status is discussed in the Supplementary Material.

**Fig. 3.**
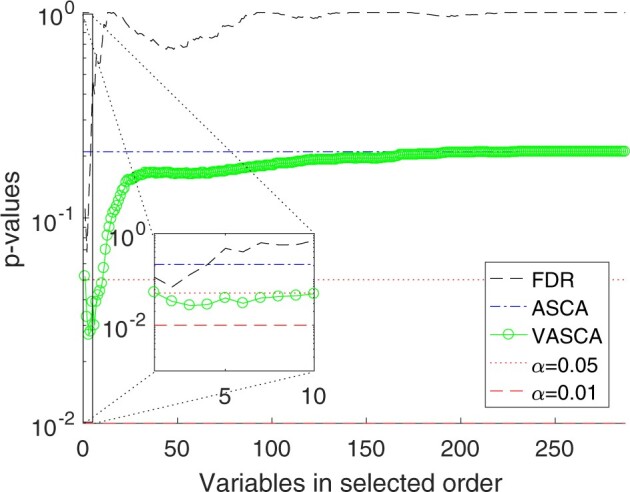
Comparison of *P*-values computed with FDR, ASCA and VASCA for the BIOASMA dataset (persistent asthma versus the rest). For the FDR, we represent the *P*-values in increasing order from left to right (from the most to the least significant variable), corrected following the procedure of BH. Whenever a corrected *P*-value exceeds 1, a value of 1 is used instead. For ASCA, a single *P*-value is shown, corresponding to the *P*-value for the complete dataset with 287 variables. For VASCA, the *P*-value at each number of variables *m* represents the significance of the dataset including the most significant *m* variables. The inset represents a detail for the first (most significant) 10 variables. Control limits of statistical significance (α=0.05 and α=0.01) are also represented

We analyze the asthma severity in Figure 3 following the same approach as in the simulated data, i.e. we compare the ordered *P*-values obtained for FDR and VASCA, and the single *P*-value for ASCA. Control limits highlighting significance for a *P*-value < 0.05 and a *P*-value < 0.01 are also shown, and the vertical axes are in logarithmic scale. Supplementary Figure S12 illustrates the results when we consider the three classes (occasional, frequent and persistent asthma), and Figure 3 when we consider persistent asthma versus the rest. In both situations, ASCA is in agreement with PLS-DA showing no statistical significance. VASCA is in agreement with sPLS-DA and significance is only found for a sub-set of variables when two classes (persistent asthma versus the rest) are considered. The FDR method fails to find statistical significance, arguably as a consequence of not directly accounting for the multivariate nature of the investigated data.

**Fig. 4.**
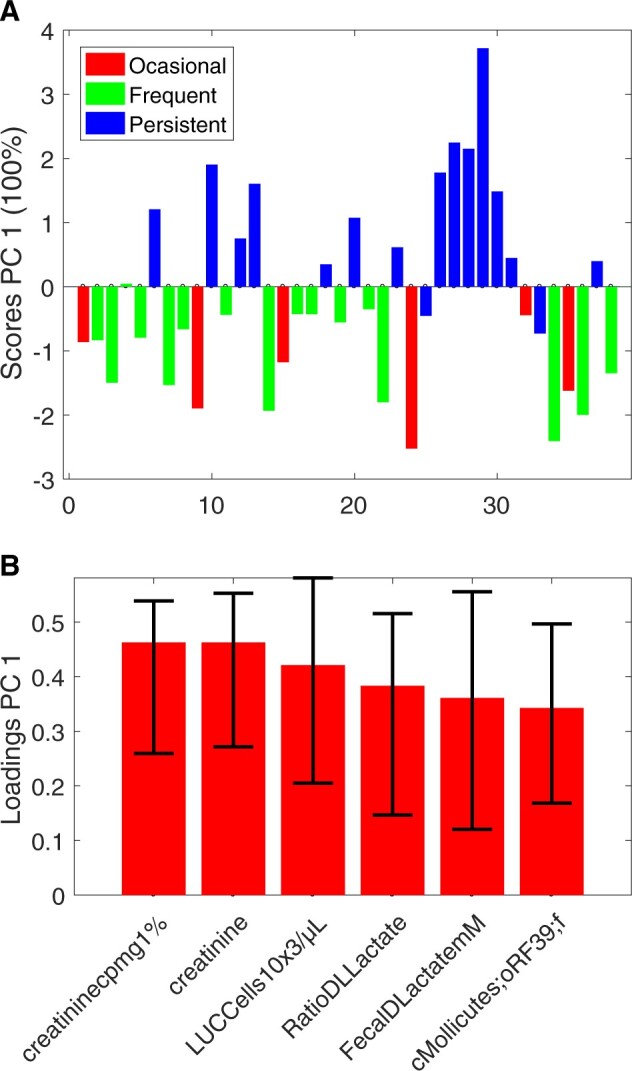
VASCA (six variables) scores (**a**) and loadings (**b**) plots for the BIOASMA dataset (persistent asthma versus the rest)

Figure 4 shows the scores and loadings for the single component in the VASCA model, which should be compared to the corresponding sPLS-DA biplots in Supplementary Figure S11. In the VASCA model, all variables present loadings which are significantly different to 0 (*P*-value < 0.05). Looking at the scores, both sPLS-DA and VASCA show similar separation ability, but VASCA selects only half of the analyzed variables, those in the left part of Supplementary Figure S11.

To provide a numerical assessment of the variable selection by sPLS-DA and VASCA, we compute the AUROC of the reduced PLS-DA models constructed on the variables selected by each method. Note that these AUROC values are expected to be overoptimistic, since they are computed from the same data used for variable selection. Yet, they are useful to compare the ability of both methods (sPLS-DA and VASCA) to find potentially interesting biomarkers. We obtain 0.99 ± 0.01 for sPLS-DA with 12 selected variables and 0.93 ± 0.02 for VASCA with 6 selected variables. We also checked the significance of the 12 variables selected by sPLS-DA using ASCA+bootstrapping in Supplementary Figure S13. We can see that only the loadings of the 6 variables originally identified by VASCA are indeed significantly different from 0. Thus, while it might be beneficial from the multivariate perspective to include the entire set of 12 variables, VASCA seems to yield a more accurate selection for the reduction of the complexity of the problem at hand.

The same analysis with the weight factor (normo-weight, overweight and obese) can be found in the Supplementary Material (see Supplementary Figs S14 and S15). In this case, ASCA does not find any significant model (just like PLS-DA) while both FDR and VASCA find a single significant variable. We obtain 0.73 ± 0.02 for PLS-DA with the three variables selected by sPLS-DA (Gomez-Llorente *et al.*, 2020) and 0.74 ± 0.01 for PLS-DA with the individual variable selected by VASCA/FDR. We finally checked the significance of the three variables selected by sPLS-DA using ASCA+bootstrapping in Supplementary Figure S16. In this case, only two of these variables (including the one originally identified by VASCA) are found to exhibit loadings significantly different from 0.

The previous analysis illustrates that both VASCA and sPLS-DA represent very interesting approaches for the Bioinformatics community, and that, beyond its inference and variable selection capabilities, VASCA can be a competitive exploratory tool for, e.g. biomarker identification.

### 5.2 Multi-factor models

In this section, we consider simultaneously the two factors of the BIOASMA dataset in a single analysis round. Supplementary Figure S17 shows the comparison of the *P*-values returned by ASCA, FDR and VASCA for two levels in each factor: persistent asthma versus the rest (occasional and frequent asthma) and normo-weight versus the rest (overweight and obese). No statistically significant results were obtained accounting simultaneously for the three levels in any of the factors. For two factors and two levels (one class versus the rest), only VASCA found statistically significant results, and with the single multi-factor model we obtain the same findings of the previous factor-wise analyses. sPLS-DA also failed in finding statistically significant results. An advantage of VASCA over (s)PLS-DA is its flexibility to handle uncorrelated factors in a single model, simplifying the analysis of complex experimental designs.

## 6 Conclusion

In this article, we presented VASCA, an extension of ANOVA Simultaneous Component Analysis (ASCA) that improves the statistical inference of multivariate models through variable selection. VASCA is inspired by the popular BH step-up procedure. Its benefits are two-fold: first, by taking on the idea of variable selection from FDR-controlling procedures, it attains substantially improved discrimination (detection) power over conventional ASCA; and second, based on multivariate inference (similar to ASCA), it is able to model/capture and visualize inherent multivariate relationships within the experimental data. Our results showed that VASCA can outperform both the BH (FDR-controlling) procedure and ASCA in terms of statistical power, and that it represents a competitive exploratory approach in comparison to widely used techniques such as PLS-DA and its sparse counterpart.

## Supplementary Material

btac795_Supplementary_Data

## Data Availability

The simulated data underlying this article are available in the repository at https://github.com/josecamachop/VASCA/tree/v1.0.0 (https://doi.org/10.5281/zenodo.7410623). The BIOASMA data underlying this article will be shared on reasonable request to the corresponding author.
