# Supplementary material for: Variable-selection ANOVA Simultaneous Component Analysis (VASCA)
Source: Bioinformatics. 2022 Dec 10;39(1):btac795. doi: 10.1093/bioinformatics/btac795 (PMC9825241; doi:10.1093/bioinformatics/btac795)
Supplement: btac795_Supplementary_Data [file btac795_supplementary_data.pdf]

Supplementary Material: Variable-Selection ANOVA Simultaneous Component Analysis (VASCA)

José Camacho[1,*], Raffaele Vitale[2], David Morales-Jimenez[1], Carolina Gómez-Llorente[3]

1. ASCA example

In this motivating example we use the data collected in [4], to which we address the readers for more details on the design of the study and the experimental techniques resorted to. Data were downloaded from the MetaboLights metabolomics public data repository (www.ebi.ac.uk/metabolights, with accession number MTBLS112). Experiments aimed at identifying changes in the metabolome of wheat (*Triticum aestivum*) induced by deoxynivalenol (DON), a mycotxin produced by the infestant *Fusarium graminearum* and related species causing the devastating plant disease Fusarium head blight. In the study, four wheat genotypes with known varying resistance to Fusarium were treated with either DON or water control and harvested at 0, 12, 24, 48 and 96 hours after treatment. Target GC-MS profiling was used to quantify an array of 57 metabolites. The resulting data matrix $\mathbf{X}$ has dimensions 296×57.

The example is included in the software repository of the paper at `https://github.com/josecamachop/VASCA/tree/v1.0.0` (DOI 10.5281/zenodo.7410623), file 'RunWheat.m'.

1.1. Factorization of the data

We postulate a model with factors time (A), trait/genotype (B) and treatment (C), with 5, 4 and 2 levels, respectively, and no interactions among factors:

$$\mathbf{X} = \mathbf{1}\mathbf{m}^{\mathrm{T}} + \mathbf{A} + \mathbf{B} + \mathbf{C} + \mathbf{E} \tag{1}$$

The coding matrix, built using deviation coding, can be inspected from the MATLAB command line by typing *paranovao.D* after the script is run.

*Corresponding author: josecamacho@ugr.es

[1]Signal Theory, Networking and Communications Department, University of Granada, C/ Periodista Daniel Saucedo Aranda s/n 18071, Granada, Spain

[2]Univ. Lille, CNRS, LASIRE (UMR 8516), Laboratoire Avancé de Spectroscopie pour les Interactions, la Réactivité et l'Environnement, F-59000, Lille, France

[3]Department of Biochemistry and Molecular Biolgoy II, School of Pharmacy, Institute of Nutrition and Food Technology "José Mataix", Biomedical Research Center, University of Granada 18160, Granada, Spain. ibs.GRANADA, Instituto de Investigación Sanitaria, 18012, Granada, Spain. CIBEROBN (Physiopathology of Obesity and Nutrition CB12/03/30038), Instituto de Salud Carlos III, 28029, Madrid, Spain

1.2. Statistical significance testing

The ANOVA-like table obtained after factorization and significance testing can be inspected from the MATLAB command line by writing: *table*. Results are displayed in Fig. S1 (mind that **X** was here auto-scaled, *i.e.*, its columns were mean-centered and normalized to unit variance) and show that all factors are statistically significant (p-value < 0.01). According to the mean squared variance captured by each ASCA submatrix (MeanSq), the most relevant factor is treatment (Factor 3).

Source	SumSq	PercSumSq	df	MeanSq	F	Pvalue
'Mean'	61649	84.23	1	61649	NaN	NaN
'Factor 1'	2013.4	2.7508	4	503.34	13.87	0.000999
'Factor 2'	1366.8	1.8674	3	455.59	12.554	0.000999
'Factor 3'	1230.4	1.6811	1	1230.4	33.904	0.000999
'Residuals'	6931.5	9.4704	191	36.291	NaN	NaN
'Total'	73191	100	200	365.96	NaN	NaN

Figure S1: ANOVA-like table returned by ASCA.

.

1.3. Visualization

For any factor/interaction, we can compute as many PCs as the corresponding degrees of freedom. The ASCA scores and loadings along the first 2 principal components (PCs) of the time submatrix are shown in Fig. S2. The red dots mark the initial time point of the longitudinal study. Time evolution follows a clockwise trend over the two-dimensional graph. Metabolites of higher abundance at each time step are located in the corresponding position within the loading plot.

The ASCA scores and loadings along the first 2 PCs of the trait submatrix are shown in Fig. S3. As one can easily see, the wheat genotype CM presents the most differential behaviour, producing less, *e.g.*, malonic acid and more, *e.g.*, citric acid.

The ASCA scores and loadings along the only PC retrievable from the treatment submatrix are shown in Fig. S4. A clear difference between treatments (DON and water control) can be spotted. Negative loadings correspond to metabolites with higher abundance in untreated samples, *e.g.*, number 44 (D-glucose) and 48 (Fructose).

There are two main benefits that ASCA yields compared to standard PCA: i) the ASCA factorization enables statistical inference as in ANOVA and ii) ASCA scores and loadings can be interpreted in the light of the unique influence of the corresponding factor/interaction.

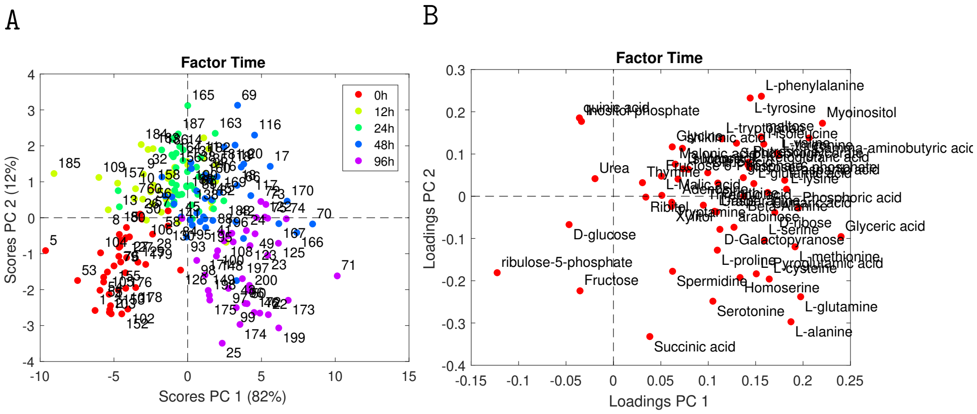

Figure S2: PCA visualization (2 PCs) of Factor Time: scores (a) and loadings (b)

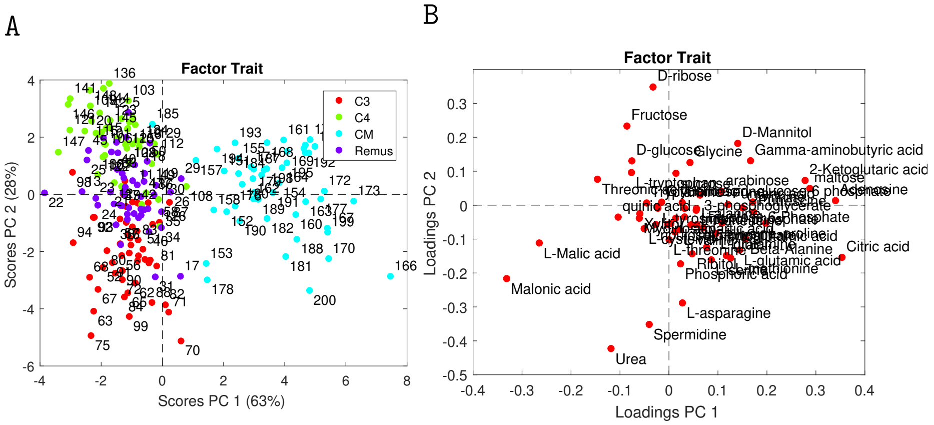

Figure S3: PCA visualization (2 PCs) of Factor Trait: scores (a) and loadings (b)

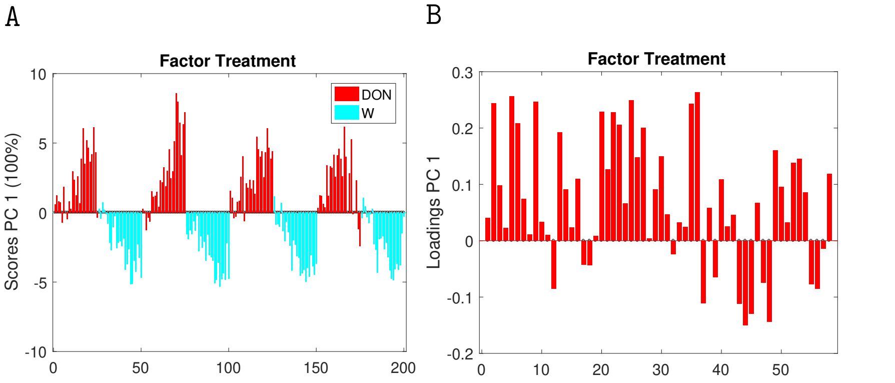

Figure S4: PCA visualization of Factor Treatment: scores (a) and loadings (b)

2. Evaluation in simulation examples

2.1. *Example 1: Non-significant relationship*

The results of this experiment are shown in Figure S5. The figure presents the ordered p-values obtained by FDR and VASCA, and the single p-value (for the complete matrix $\mathbf{X}$) returned by ASCA. Average results are shown with the corresponding lines and the shadowed areas represent standard deviations. Control limits highlighting significance for a p-value < 0.05 and a p-value < 0.01 are also displayed. Note the vertical axes are in logarithmic scale. The figure shows that all methods yield p-values well above the control limits, illustrating their robustness against Type-I errors. QQ-plots for ASCA and VASCA (Fig. S6) show that the p-values are uniformly distributed under the null-hypothesis, as expected.

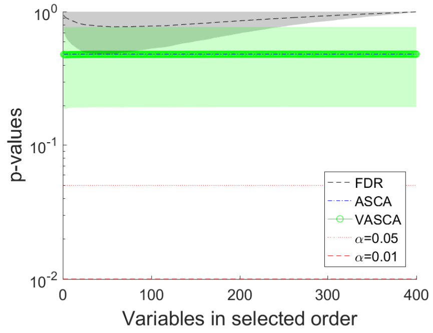

Figure S5: Example 1: No relationship between $\mathbf{X}$ and $\mathbf{C}$. Comparison of p-values computed with FDR, ASCA and VASCA. For each method, average p-values from 1000 simulations are shown together with the area between average +/- one standard deviation. For the FDR, we represent the p-values in increasing order from left to right (from the most to the least significant variable), corrected following the procedure of Benjamini-Hochberg (BH). Whenever a corrected p-value exceeds 1, a value of 1 is used instead. For ASCA, a single p-value is shown, corresponding to the p-value for the data set with 400 variables averaged over the 1000 simulations. For VASCA, the p-value at each number of variables m represents the significance of the data set including the most significant m variables. Control limits of statistical significance ($\alpha = 0.05$ and $\alpha = 0.01$) are also represented.

2.2. *Example 2: Significant one-to-one relationships*

Scores and loadings of the VASCA model are shown in Figure S7. The scores (left) clearly distinguish the two design levels confirming the significance of the model. The loadings (right) show that 3 variables are by far the most relevant of the 6 under study. If we derive bootstrapping intervals, we can see that only the loadings for those three variables are significantly different from 0.

In Table S1 we show the percentage of simulations where at least 1, 2 and 3 of the significant variables were found to be statistically significant along with the FPR. The results are satisfactory for the three compared methods, although VASCA shows an increase in the FPR if we compute it including all variables in

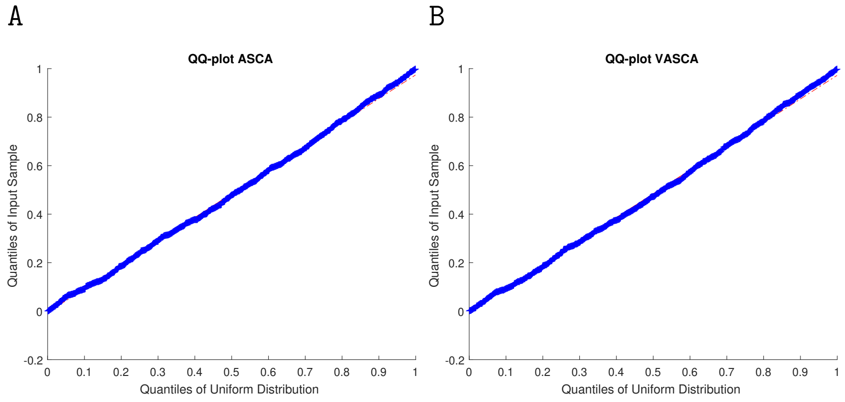

Figure S6: Example 1: QQ-plot showing that p-values are uniformly distributed under the null-hypothesis for ASCA (A) and VASCA (B–first most significant variable).

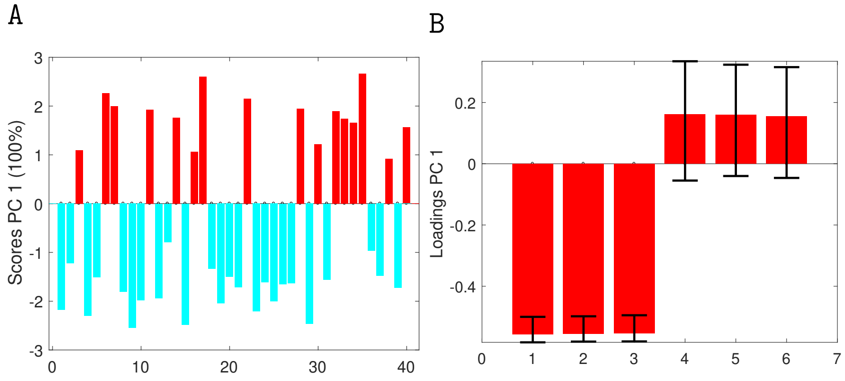

Figure S7: Example 2: VASCA (6 variables) scores (a) and loadings (b) plots. Simulation with one-to-one relationships between 3 variables in $\mathbf{X}$ and $\mathbf{C}$.

significant matrices. If we apply bootstrapping along with VASCA, we reduce the FPR to a reasonable (but still slightly overoptimistic) level.

The result for the case of one-to-one relationships between 3 variables in $\mathbf{X}$ and $\mathbf{C}$ generated with a small bias is shown in Figure S8. We can see that the FDR does not detect a significant relationship on average anymore. VASCA, however, shows a higher statistical power and determines on average a p-value < 0.05 for the most significant variable. In Table S2 we show that VASCA (without and with bootstrapping) outperforms the FDR while controlling the FPR.

Finally, we also measured computation times for the different algorithmic procedures (including permutation testing) in the Matlab environment. The ASCA analysis of a dataset with 40 observations from a single simulation takes below the second on a regular computer. The VASCA analysis of the same dataset takes few seconds, while the FDR and VASCA + bootstrapping assess-

Table S1: Example 2: One-to-one relationships between 3 variables in **X** and **C**. Proportion of simulations where at least 1, 2 and 3 of the significant variables (as predefined in the experiment) were found to be statistically significant by the different methods, and Type-I Error measured as False Positive Rate (FPR). Comparison of FDR and VASCA (without and with bootstrapping) for a significance level of 0.01.

Method	1 variable	2 variables	3 variables	FPR
FDR	1.00	1.00	1.00	$4.7x10^{-4}$
VASCA	1.00	1.00	1.00	0.062
VASCA + bootstrap	1.00	1.00	1.00	0.023

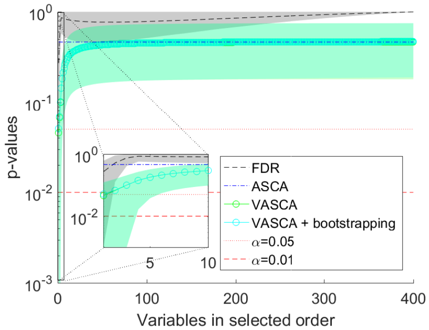

Figure S8: Example 2b: One-to-one relationships between 3 variables in **X** and **C** generated with a smaller bias. Comparison of p-values computed with FDR, ASCA and VASCA (without and with bootstrapping). For each method, average p-values from 1000 simulations are shown together with the area between average +/- one standard deviation. For the FDR, we represent the p-values in increasing order from left to right (from the most to the least significant variable), corrected following the procedure of Benjamini-Hochberg (BH). Whenever a corrected p-value exceeds 1, a value of 1 is used instead. For ASCA, a single p-value is shown, corresponding to the p-value for the data set with 400 variables averaged over the 1000 simulations. For VASCA, the p-value at each number of variables m represents the significance of the data set including the most significant m variables. Control limits of statistical significance ($\alpha = 0.05$ and $\alpha = 0.01$) are also represented.

ments below the minute.

2.3. Example 3: Multivariate relationship

In Table S3 we show the percentage of simulations where at least 1, 2 and 3 of the significant variables were found to be statistically significant along with the FPR. The results show the increased power of VASCA over the FDR in a similar way as in the previous example.

2.4. Example 4: Multivariate relationship in two factors and interaction with several levels

The comparison results are presented in Figure S9. To some extent, the outcomes resemble those of Example 3 in the main manuscript, with an increased power of VASCA in comparison to FDR. Moreover, ASCA-genes, returns averaged p-values generally lower than VASCA ones for non-significant variables,

Table S2: Example 2b: One-to-one relationships between 3 variables in **X** and **C** generated with a smaller bias. Proportion of simulations where at least 1, 2 and 3 of the significant variables (as predefined in the experiment) were found to be statistically significant by the different methods, and Type-I Error measured as False Positive Rate (FPR). Comparison of FDR and VASCA (without and with bootstrapping) for a significance level of 0.01.

Method	1 variable	2 variables	3 variables	FPR
FDR	0.22	0.04	0.003	$3.0x10^{-4}$
VASCA	0.43	0.24	0.07	0.01
VASCA + bootstrap	0.43	0.24	0.07	0.003

Table S3: Example 3: Multivariate relationship between 3 variables in **X** and **C**. Proportion of simulations where at least 1, 2 and 3 of the significant variables (as predefined in the experiment) were found to be statistically significant by the different methods, and Type-I Error measured as False Positive Rate (FPR). Comparison of FDR and VASCA (without and with bootstrapping) for a significance level of 0.01.

Method	1 variable	2 variables	3 variables	FPR
FDR	0.23	0.01	0	$1.4x10^{-5}$
VASCA	0.47	0.20	0.04	0.009
VASCA + bootstrap	0.47	0.20	0.03	0.002

A B

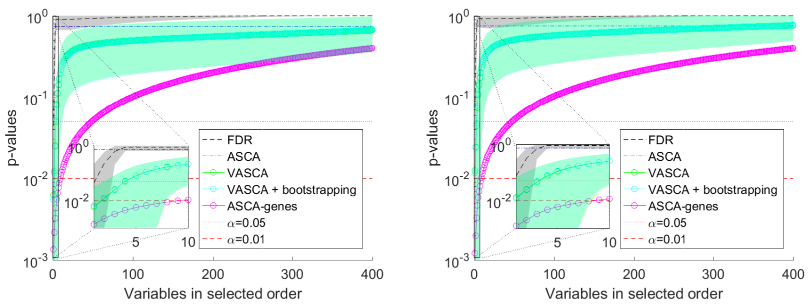

Figure S9: Example 4: Multivariate relationship between 3 variables in **X** and **C** for a design with 2 factors of 4 and 3 levels, respectively. Factor 1 (a) and 2 (b). Comparison of p-values computed with FDR, ASCA and VASCA (without and with bootstrapping). For each method, average p-values from 1000 simulations are shown together with the area between average +/- one standard deviation. For the FDR, we represent the p-values in increasing order from left to right (from the most to the least significant variable), corrected following the procedure of Benjamini-Hochberg (BH). Whenever a corrected p-value exceeds 1, a value of 1 is used instead. For ASCA, a single p-value is shown, corresponding to the p-value for the data set with 400 variables averaged over the 1000 simulations. For VASCA, the p-value at each number of variables m represents the significance of the data set including the most significant m variables. For ASCA-genes, the p-values correspond to those obtained based on model leverage. The inset represents a detail for the first (most significant) 10 variables. Control limits of statistical significance ($\alpha = 0.05$ and $\alpha = 0.01$) are also represented.

behaviour potentially connected with the increased Type-I error the former suffers from.

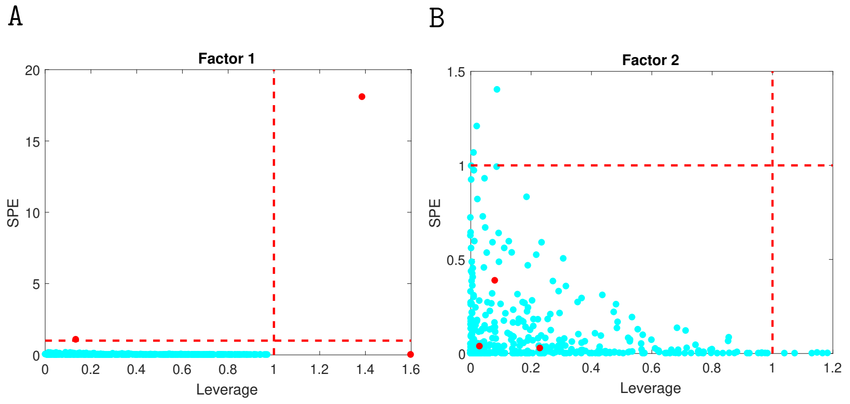

Figure S10: Variable selection by the ASCA-genes method. Factor 1 (a) and 2 (b). Ground-truth significant variables are represented in dark (red) color. Control limits of statistical significance ($\alpha = 0.01$) are represented as dashed lines.

We illustrate the results yielded by the ASCA-genes method [2] in Figure S10. Two of the relevant variables (marked in dark red color) varying with Factor 1 show high leverage. The other significant variable is found in the SPE chart. For factor 2, however, there are several non-significant variables that exceed the leverage and SPE control limits, which should be regarded as false positives. In this specific example, VASCA detected two significant variables for Factor 1 (the third presented a p-value close to 0.05) and none for Factor 2, FDR could only detect one significant variable for Factor 1, and VASCA + bootstrap none.

3. Results on real data

3.1. Model for asthma severity

A sPLS-DA model with 12 variables was found statistically significant [1] to distinguish the persistent asthma class from the rest (occasional and frequent asthma) with an Area Under the Receiver Operating Characteristics curve (AUROC) of 0.66 ± 0.08 (p-value < 0.05) in double cross-validation [3], and a sPLS-DA model with three variables was found statistically significant to distinguish the normo-weight class from the rest (overweight and obese) with an AUROC of 0.75 ± 0.09 (p-value < 0.05). The PLS-DA model for asthma severity with only selected variables is presented in Figure S11, where the scores show the clear separation between persistent asthma and the rest.

We analyze the asthma severity in Figure S12 following the same approach as for the simulated data, comparing the ordered p-values resulting from FDR and VASCA, and the single p-value returned by ASCA. Control limits highlighting significance for a p-value < 0.05 and a p-value < 0.01 are also displayed, and the vertical axes are in logarithmic scale. Figure S12 illustrates the results when we consider the three classes (occasional, frequent and persistent asthma).

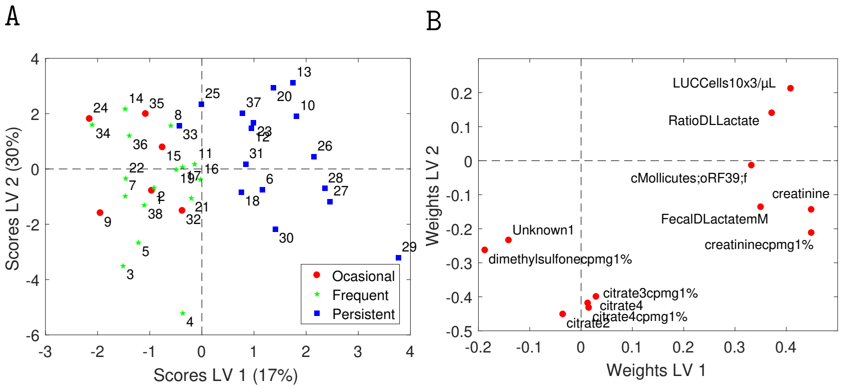

Figure S11: PLS-DA model for class "persistent asthma" vs the rest (occasional and frequent asthma) in the BIOASMA data set obtained in [1]: (a) scores plot and (b) loadings plot.

In Figure S13 we show the VASCA loading plot (with bootstrapping-derived confidence intervals) resulting from the analysis of a reduced version of the BIOASMA dataset containing only the 12 variables selected by sPLS-DA. We can see that only the 6 variables originally identified by VASCA show loadings significantly different from 0.

3.2. Model for weight classification

The PLS-DA model for weight classification with only selected variables is presented in Figure S14, where the scores show the separation between normo-weight and the rest.

We analyze the weight classification in Figure S15 following the same approach as before. Figure S15(a) illustrates the results when we consider the three classes (normo-weight, overweight and obese), and Figure S15(b) when we consider normo-weight vs the rest. In both situations, ASCA is in agreement with PLS-DA showing no statistical significance. VASCA and FDR are in agreement with sPLS-DA and significance is only found for a sub-set of variables when two classes (normo-weight class vs the rest) are considered.

In Figure S16 we show the VASCA loadings plot (with bootstrapping-derived confidence intervals) resulting from the analysis of a reduced version of the BIOASMA dataset containing only the 3 variables selected by sPLS-DA. We can see that only 2 of these variables (including the one originally identified by VASCA) are found to exhibit loadings significantly different from 0.

3.3. Multi-factor model

In this section we consider simultaneously the two factors of the BIOASMA data set in a single analysis. Figure S17 shows the comparison of p-values for ASCA, FDR and VASCA taking into account two levels within each factor: persistent asthma vs the rest (occasional and frequent asthma) and normo-weight vs the rest (overweight and obese). No significant results were obtained when all three levels are considered within the two factors.

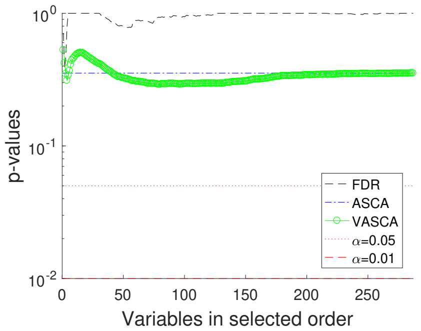

Figure S12: Comparison of p-values computed with FDR, ASCA and VASCA for the BIOASMA data set (occasional, frequent and persistent asthma). For the FDR, we represent the p-values in increasing order from left to right (from the most to the least significant variable), corrected following the procedure of Benjamini-Hochberg (BH). Whenever a corrected p-value exceeds 1, a value of 1 is used instead. For ASCA, a single p-value is shown, corresponding to the p-value for the data set with 287 variables. For VASCA, the p-value at each number of variables m represents the significance of the data set including the most significant m variables. Control limits of statistical significance ($\alpha = 0.05$ and $\alpha = 0.01$ are also represented.

References

[1] Gomez-Llorente, M. *et al.* (2020). A multi-omics approach reveals new signatures in obese allergic asthmatic children. *Biomedicines*, **8**(9), 359.

[2] Nueda, M. J. *et al.* (2007). Discovering gene expression patterns in time course microarray experiments by anova–sca. *Bioinformatics*, **23**(14), 1792–1800.

[3] Szymańska, E. *et al.* (2012). Double-check: validation of diagnostic statistics for PLS-DA models in metabolomics studies. *Metabolomics*, **8**(1), 3–16.

[4] Warth, B. *et al.* (2014). Gc–ms based targeted metabolic profiling identifies changes in the wheat metabolome following deoxynivalenol treatment. *Metabolomics*, **11**(3), 722–738.

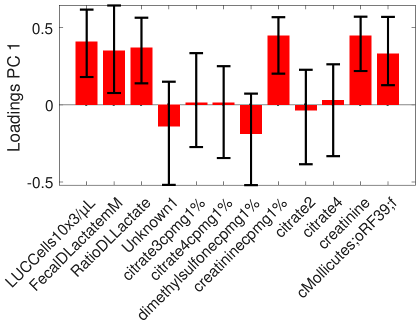

Figure S13: VASCA loading plots with bootstrapping-derived confidence intervals for the BIOASMA data set (persistent asthma vs the rest) and the 12 variables selected by sPLS-DA.

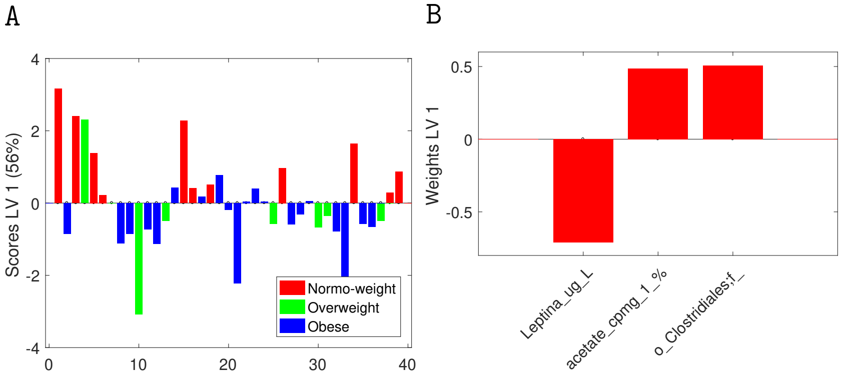

Figure S14: PLS-DA model for class "persistent asthma" vs the rest (occasional and frequent asthma) in the BIOASMA data set obtained in [1]: (a) scores plot and (b) loadings plot.

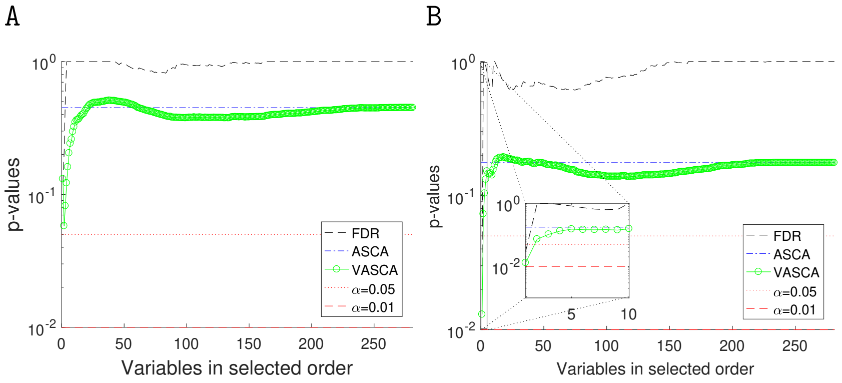

Figure S15: Comparison of p-values computed with FDR, ASCA and VASCA for the BIOASMA data set–(a) normo-weight vs overweight vs obese and (b) normo-weight vs the rest. For the FDR, we represent the p-values in increasing order from left to right (from the most to the least significant variable), corrected following the procedure of Benjamini-Hochberg (BH). Whenever a corrected p-value exceeds 1, a value of 1 is used instead. For ASCA, a single p-value is shown, corresponding to the p-value for the data set with 287 variables. For VASCA, the p-value at each number of variables m represents the significance of the data set including the most significant m variables. The inset represents a detail for the first (most significant) 10 variables. Control limits of statistical significance ($\alpha = 0.05$ and $\alpha = 0.01$ are also represented.

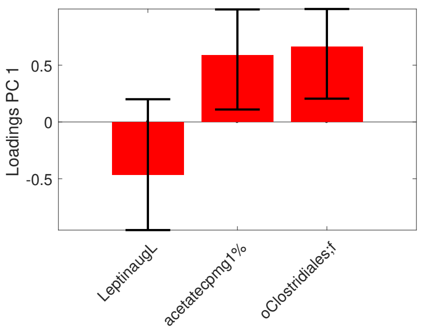

Figure S16: VASCA loading plots with bootstrapping-derived confidence intervals for the BIOASMA data set (normo-weight vs the rest) and the 3 variables selected by sPLS-DA.

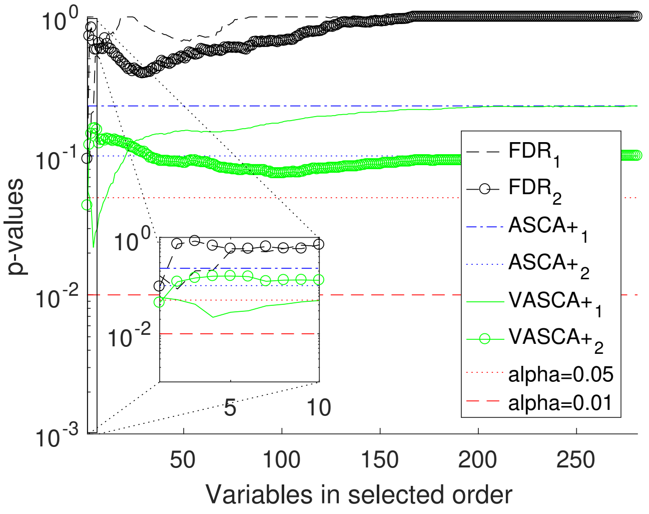

Figure S17: Comparison of p-values computed with FDR, ASCA and VASCA. For the FDR, we represent the p-values in increasing order from left to right (from the most to the least significant variable), corrected following the procedure of Benjamini-Hochberg (BH). Whenever a corrected p-value exceeds 1, a value of 1 is used instead. For ASCA, a single p-value is shown, corresponding to the p-value for the data set with 287 variables. For VASCA, the p-value at each number of variables m represents the significance of the data set including the most significant m variables. The inset represents a detail for the first (most significant) 10 variables. Control limits of statistical significance ($\alpha = 0.05$ and $\alpha = 0.01$) are also represented.
